# In response to ‘Integrated ultrasound haemodynamic profiling (VTI-VExUS) for risk stratification in acute heart failure’

**DOI:** 10.1093/ehjimp/qyag072

**Published:** 2026-04-16

**Authors:** Guilherme Pinheiro Machado, Guilherme Heiden Telo, Scheila Signor, Gustavo Neves de Araujo, Marco Vugman Wainstein

**Affiliations:** Department of Cardiology, Hospital de Clínicas de Porto Alegre, Porto Alegre, Brazil; Department of Cardiology, Hospital de Clínicas de Porto Alegre, Porto Alegre, Brazil; Department of Cardiology, Hospital de Clínicas de Porto Alegre, Porto Alegre, Brazil; Department of Cardiology, Hospital Unimed Grande Florianópolis, Florianópolis, Brazil; Department of Cardiology, Hospital de Clínicas de Porto Alegre, Porto Alegre, Brazil

**This Letter refers to ‘Integrated ultrasound haemodynamic profiling (VTI–VExUS) for risk stratification in acute heart failure', by O. I. Ruiz–Fuentes**  ***et al.,***  **https://doi.org/10.1093/ehjimp/qyag012.**

Dear Editor,

We read with great interest the recent article by Ruiz-Fuentes *et al*.^1^ on integrated ultrasound haemodynamic profiling (VTI–VExUS) in acute heart failure (AHF). The authors should be congratulated for proposing a physiologically coherent framework integrating systemic congestion and forward flow. Their findings reinforce the limitations of traditional bedside evaluation and highlight that point-of-care ultrasound (POCUS) enhances clinical assessment beyond the physical examination. In this context, the improvement in discrimination observed with VTI–VExUS [area under the curve (AUC) = 0.74 vs. 0.58 for clinical assessment] highlights the clinical relevance of ultrasound-based assessment.

We wish to share complementary data from our ST-elevation myocardial infarction (STEMI) cohort that both validates their approach in a distinct, high-acuity setting and offers a perspective on which ultrasound tool may be most appropriate depending on the clinical context. In a cohort of 185 patients with STEMI, we applied the VTI–VExUS classification as a validation approach in our cohort, alongside previously established ultrasound-based strategies, including VExUS alone and the LUV classification. Importantly, VExUS^2^ had been previously validated in our cohort, whereas the LUV^3^ was originally derived from it. Briefly, the VExUS (Venous Excess Ultrasound Score) is a bedside tool used to assess systemic venous congestion by integrating inferior vena cava (IVC) size with Doppler patterns of the hepatic, portal, and intrarenal veins, allowing classification into grades from no/mild (0–1) to severe congestion (grade 3). The VTI–VExUS approach combines this assessment of congestion with evaluation of forward flow using the left ventricular outflow tract velocity-time integral (LVOT-VTI). The LUV classification integrates **Lu**ng ultrasound and LVOT-**V**TI for risk stratification in STEMI. Patients are stratified into four profiles (A-D) based on pulmonary congestion (defined as ≥3 positive lung ultrasound zones) and LVOT-VTI values (≤14 cm); (A: no pulmonary congestion/normal flow; B: congestion/normal flow; C: no congestion/low flow; D: congestion/low flow). Notably, the LVOT-VTI threshold adopted in the VTI–VExUS classification was derived from this previously established framework.

This analysis was from a single-centre prospective cohort. The mean age of the population was 61.9 ± 11 years, and 55% were female. Anterior STEMI was present in 43% of patients, whereas 13% were classified as Killip class III–IV at presentation. The rate of in-hospital mortality was 7.6%. The primary outcome in our analysis was in-hospital mortality, which reflects the acute severity of STEMI and differs from the 30-day composite endpoint used by Ruiz-Fuentes *et al*. tailored to AHF populations. Among the three tools evaluated, the LUV classification demonstrated the highest discriminative performance (AUC = 0.924), followed by VTI–VExUS (AUC = 0.847), and VExUS alone (AUC = 0.662) (*Figure 1*). These findings reinforce that integrated ultrasound approaches enhance risk stratification beyond isolated parameters. They also suggest that POCUS consistently outperforms clinical evaluation alone and that the optimal combination of variables may vary according to the clinical scenario.

**Figure 1:**
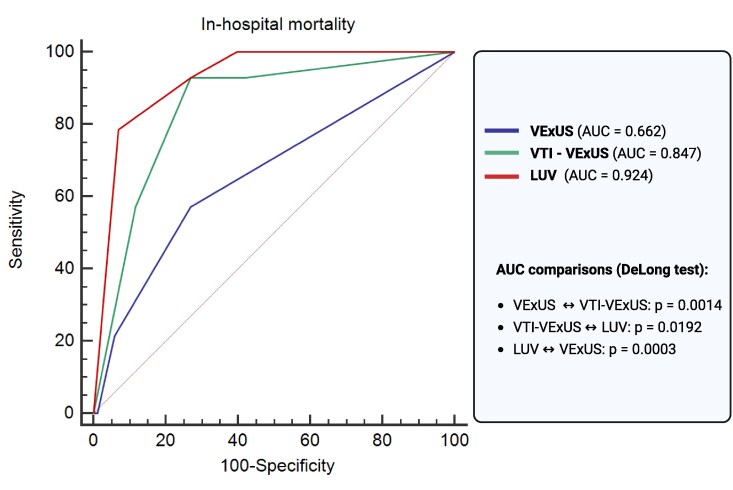
Receiver operating characteristic curves of VExUS, VTI-VExUS, and classifications to predict in-hospital mortality of patients admitted with STEMI and comparisons with DeLong’s test. Figure Created in https://BioRender.com

The study by Ruiz-Fuentes *et al*.^1^ also included patients with AHF triggered by acute coronary syndromes, such as STEMI. However, STEMI represents a distinct haemodynamic phenotype, in which early pulmonary congestion—captured by lung ultrasound—may play a more prominent role than systemic venous congestion. Importantly, lung congestion is not absent in right ventricular myocardial infarction, as demonstrated by Araujo *et al*.,^4^ who showed that a substantial proportion of patients with RV STEMI exhibit pulmonary congestion on admission lung ultrasound, challenging the traditional concept of ‘clear lungs’ in this setting. Nevertheless, the VTI–VExUS framework remains highly valuable. By integrating congestion and perfusion, it offers a comprehensive haemodynamic assessment that may be particularly useful in scenarios where systemic congestion predominates. For example, in right ventricular myocardial infarction or conditions characterized by elevated right-sided pressures, VExUS-derived parameters may provide incremental prognostic insight beyond pulmonary evaluation alone.

Taken together, these data support three key considerations. First, POCUS enhances clinical evaluation, providing objective haemodynamic information that improves early risk stratification. Second, an integrated approach is superior to single-parameter approaches, regardless of whether congestion or perfusion is assessed in isolation. Third, the optimal ultrasound strategy is context-dependent, with lung ultrasound–based approaches potentially outperforming venous Doppler in predominantly left-sided syndromes such as STEMI, whereas VExUS-based approaches may be more informative in right-sided or systemic congestion states.

In addition, POCUS can support decision-making regarding mechanical circulatory support (MCS), as illustrated by Fortuni *et al*.^5^ VExUS enables quantification of systemic venous congestion, identifying patients with significant backward failure and end-organ involvement, which may influence the timing of escalation and guide decongestive strategies. The addition of LVOT-VTI to the VExUS approach provides an assessment of forward flow, facilitating the identification of patients with the high-risk ‘systemic congestion plus low-flow’ phenotype, who may derive the greatest benefit from early MCS. Similarly, the LUV classification integrates pulmonary congestion with LVOT-VTI, enabling rapid bedside stratification into profiles that reflect distinct haemodynamic states, with the LUV D profile (lung congestion and low flow) representing a particularly high-risk subgroup. Importantly, these approaches enable a more refined assessment and support a more individualized strategy for MCS use in high-risk patients.

However, future studies should directly compare these multimodal ultrasound strategies across different acute cardiovascular conditions, ideally using standardized endpoints and prospective validation. Moreover, whether ultrasound-guided therapeutic strategies based on these classifications can translate into improved outcomes remains an important unanswered question.

In conclusion, the work by Ruiz-Fuentes *et al*.^1^ represents an important step towards a physiology-driven approach to bedside haemodynamic assessment. Our findings extend this concept by providing external validation in a STEMI cohort, suggesting that while VTI–VExUS is a versatile tool, the LUV classification may demonstrate superior prognostic performance in this specific setting. Ultimately, tailoring POCUS-based haemodynamic profiling to the underlying pathophysiology may represent the next step toward individualized cardiovascular care.

## Data Availability

The data underlying this article will be shared on reasonable request to the corresponding author.

## Lead author biography

Guilherme Pinheiro Machado, MD, MSc, PhD, is an interventional cardiologist from Porto Alegre, Brazil. He completed his training in Cardiology and Interventional Cardiology at the Hospital de Clínicas de Porto Alegre (HCPA), a tertiary academic centre affiliated with the Federal University of Rio Grande do Sul (UFRGS), where he also obtained his Master’s degree and PhD in Cardiovascular Sciences. Dr Machado has over a decade of experience in cardiovascular research, with a particular focus on risk stratification in acute coronary syndromes, point-of-care ultrasound (POCUS), and haemodynamic assessment. He has authored multiple peer-reviewed publications in high-impact journals. Recently, he was selected for a fellowship in intracoronary imaging and coronary physiology at Imperial College London, in partnership with the Brazilian Society of Hemodynamic and Interventional Cardiology (SBHCI).

## References

[1] Ruiz-Fuentes OI, Barron-Martinez A, Gonzalez-Macedo E, Viana-Rojas JA, Sierra-Gonzalez de Cossio A, Gopar-Nieto R et al Integrated ultrasound haemodynamic profiling (VTI–VExUS) for risk stratification in acute heart failure. Eur Heart J Imaging Methods Pract 2026;4:qyag012. 41646879 10.1093/ehjimp/qyag012 PMC12871076

[2] Machado GP, Telo GH, Barbato JPDR, Saadi MP, Araujo GN, Chies A et al Venous Excess Ultrasound (VExUS) score in patients with ST-elevation myocardial infarction to predict in-hospital mortality. Arq Bras Cardiol 2025;122:e20250093. 40736126 10.36660/abc.20250093 PMC12269907

[3] Machado GP, Telo GH, de Araujo GN, da Rosa Barbato JP, Amon A, Martins A et al A combination of left ventricular outflow tract velocity time integral and lung ultrasound to predict mortality in ST elevation myocardial infarction. Intern Emerg Med 2024;19:2167–76. 39044051 10.1007/s11739-024-03719-z

[4] de Araujo GN, Telo GH, Machado GP, da Silveira AD, Scolari FL, Nassif MP et al Right ventricular ST-segment elevation myocardial infarction and pulmonary congestion: insights from a prospective cohort of patients evaluated by admission lung ultrasound. J Am Soc Echocardiogr 2026;39:422–4. 41456686 10.1016/j.echo.2025.12.006

[5] Fortuni F, Zilio F, Iannopollo G, Ciliberti G, Trambaiolo P, Ceriello L et al Management of temporary mechanical circulatory support devices in cath-lab and cardiac intensive care unit. Eur Heart J Imaging Methods Pract 2023;1:qyad011. 39044800 10.1093/ehjimp/qyad011 PMC11195697

